# *Chlorella sorokiniana* Dietary Supplementation Increases Antioxidant Capacities and Reduces ROS Release in Mitochondria of Hyperthyroid Rat Liver

**DOI:** 10.3390/antiox9090883

**Published:** 2020-09-17

**Authors:** Gaetana Napolitano, Gianluca Fasciolo, Giovanna Salbitani, Paola Venditti

**Affiliations:** 1 Dipartimento di Scienze e Tecnologie, Università degli Studi di Napoli Parthenope, via Acton n. 38, I-80133 Napoli, Italy; gaetana.napolitano@uniparthenope.it; 2 Dipartimento di Biologia, Università di Napoli Federico II, Complesso Universitario Monte Sant’Angelo, via Cinthia, I-80126 Napoli, Italy; gianluca.fasciolo@unina.it (G.F.); giovanna.salbitani@unina.it (G.S.)

**Keywords:** microalgae, *Chlorella sorokiniana*, hyperthyroidism, oxidative damage, rat liver, mitochondria

## Abstract

The ability of aerobic organisms to cope with the attack of radicals and other reactive oxygen species improves by feeding on foods containing antioxidants. Microalgae contain many molecules showing in vitro antioxidant capacity, and their food consumption can protect cells from oxidative insults. We evaluated the capacity of dietary supplementation with 1% dried *Chlorella sorokiniana* strain 211/8k, an alga rich in glutathione, α-tocopherol, and carotenoids, to counteract an oxidative attack in vivo. We used the hyperthyroid rat as a model of oxidative stress, in which the increase in metabolic capacities is associated with an increase in the release of mitochondrial reactive oxygen species (ROS) and the susceptibility to oxidative insult. *Chlorella sorokiniana* supplementation prevents the increases in oxidative stress markers and basal oxygen consumption in hyperthyroid rat livers. It also mitigates the thyroid hormone-induced increase in maximal aerobic capacities, the mitochondrial ROS release, and the susceptibility to oxidative stress. Finally, alga influences the thyroid hormone-induced changes in the factors involved in mitochondrial biogenesis peroxisomal proliferator-activated receptor-γ coactivator (PGC1-1) and nuclear respiratory factor 2 (NRF-2). Our results suggest that *Chlorella sorokiniana* dietary supplementation has beneficial effects in counteracting oxidative stress and that it works primarily by preserving mitochondrial function. Thus, it can be useful in preventing dysfunctions in which mitochondrial oxidative damage and ROS production play a putative role.

## 1. Introduction

The use of oxygen as the final acceptor of the electrons in the oxidation of energy substances of food origin allows aerobic organisms to produce high amount of metabolic energy. The high aerobic organisms’ ability to produce ATP is associated with the continuous production of radicals and other reactive oxygen species (ROS) that originate as side products of oxidative metabolism.

ROS play many useful roles in cells by acting as cell signaling molecules, but they can also oxidize and damage biological macromolecules. ROS include species highly reactive (the hydroxyl radical, ^•^OH) and poorly reactive (the superoxide O_2_^•−^ and the hydrogen peroxide H_2_O_2_). They originate by aerobic processes at several cell sites, among which are peroxisomes, endoplasmic reticulum, nicotinamide adenine dinucleotide phosphate oxidase, and mitochondria. The latter are considered to be the main source, due to their high oxygen consumption [1]. Mitochondria are also the main targets of the ROS, which, in turn, can damage the mitochondrial transport chain of electrons, compromising its functionality [2]. Mitochondrial oxidative damage can trigger a positive feedback circuit in which damaged mitochondria increase their ROS release by exacerbating their damage and damaging other cellular components [3].

The cells protect themselves from oxidative damage with a complex system of antioxidants that act at different levels neutralizing the ROS and so preventing their harmful actions or repairing the damage once it has arisen. The antioxidant system comprises endogenously produced enzymes and low molecular weight molecules and substances that must be assumed through food, mainly fruits and vegetables [4].

In some physio-pathological conditions, an imbalance between ROS production and antioxidant defenses system can develop. In these conditions, an oxidative stress state arises, [5] which can contribute to the development and progression of some diseases, such as metabolic and neurodegenerative diseases [1].

Some experimental evidence shows that the intake of single antioxidants is ineffective in preventing the diseases and may be harmful, with unwanted consequences for the health, mainly in well-nourished people, and that the diet is the optimal source of antioxidants [6]. This can occur, because there is a mix of molecules acting as antioxidants and having synergistic actions in antioxidant-rich foods. Therefore, the search for antioxidant-rich foods to attempt to increase antioxidant defenses is a very active field in the functional food industry.

Microalgae have great potential for applications, being rich in many compounds that have antioxidant properties such as pigments, antioxidant enzymes, and vitamins, and so their diet consumption furnishes multiple antioxidants components [7,8,9].

Among the microalgae, the Chlorella genus is one of the most exploited and consists of many unicellular subspecies distributed in both freshwater and saline environments. To date, the characterized *Chlorella* species are more than 20, with over 100 described strains. *Chlorella sorokiniana* is a small subspecies isolated in 1953 by Sorokin [10]. The reverse-phase HPLC analysis of its composition showed that the total carotenoid content is 0.69% of dry matter. The content in α tocopherol, carotene, and lutein is 112, 600, and 4300 μg/g of dry matter, respectively [11]. HPLC analysis of the *Chlorella sorokiniana* 211/8k strain confirmed the presence of high levels of carotenoids, among which are neoxanthin, β-carotene, and high levels of lutein (~60% of total carotenoids) [12]. All these compounds have high radical scavenging properties [11] and suggest such an alga as a possible source of exogenous antioxidants.

In the present paper, we evaluated the in vivo antioxidant capacity of *C. sorokiniana* 211/8k using, as a model of oxidative stress, rats made experimentally hyperthyroid. The increase of the plasma level of the thyroid hormone is associated with the increases of both the metabolic rate and the content of oxidatively damaged macromolecules in the target tissues of the hormone, including the liver [13]. The increased oxidative damage is due both to increased mitochondrial ROS release [14] and decreased antioxidant capacities [13].

For this study, *C. sorokiniana* was grown in a culture medium supplemented with bicarbonate to enhance the cell quality of the algae. In fact, according to recent studies, a bicarbonate addition to microalgae improves the growth rate and the accumulation of beneficial molecules such as pigments and lipids [15,16]. To assess the capacity of the alga to counteract the thyroid hormone-induced hepatic oxidative damage and the involvement of mitochondria, we measured the levels of lipid hydroperoxides and protein carbonyls as markers of oxidative damage to lipids and proteins, respectively. To get information on the role played by the mitochondria, we determined the tissue and mitochondrial respiration, oxidative capacities, and mitochondrial ROS release. To get information on the alga effect on antioxidant capacities, we evaluated the activities of two of the main antioxidant enzymes, glutathione peroxidase and reductase, and the in vitro susceptibility to Fe^2+^/ascorbate-induced oxidative stress. Finally, we examined the effects of the alga on the thyroid hormone-induced mitochondrial proliferation, measuring the tissue levels of the main factors regulating such a process (peroxisomal proliferator-activated receptor-γ coactivator, PGC-1, and the nuclear respiratory factors 1 and 2, NRF-1 and NRF-2, respectively).

## 2. Materials and Methods

### 2.1. Algal Strains and Growth Conditions

*Chlorella sorokiniana* Shihira and Krauss strain 211/8k (CCAP of Cambridge University) was grown in a 2-L batch at 32 ± 0.5 °C under continuous light (fluorescent lamps, 250 µmol photons/m^2^ s^1^) and flushed with air. The basal medium composition was previously reported by Salbitani et al. [17], and the pH of the medium was 6.5. Cultures of *C. sorokiniana* were grown using bicarbonate (NaHCO_3_ 1 g/L) as an inorganic carbon source. A single administration of NaHCO_3_ was made when cultures were in the lag phase (~1.5 × 10^6^ cell/mL; optical density (OD) 0.1–0.2).

*Chlorella* cultures were monitored daily, and the growth was spectrophotometrically evaluated by measuring the optical density (OD) at 800 nm. For experiments, cells were harvested at the late-exponential phase (OD_800_: 0.8) by low-speed centrifugation at 4500× *g* for 8 min. The cellular pellet was dried at 80° in hot air oven until getting a constant weight. The dried cells were then used as dietary supplementation for rats.

### 2.2. Pigment Contents

Cells from 3 mL of culture were collected by low-speed centrifugation (4000× *g* for 5–10 min). The pellets were resuspended in 3 mL of N, N-dimethylformamide and kept in the dark at 4 °C. After 12–24 h, chlorophylls and carotenoids were evaluated spectrophotometrically. For chlorophylls determination, the absorbance was measured at 647 and 664 nm, and the content was calculated according to Carfagna et al. [18]. The carotenoids amount was calculated according to Wellburn [19], measuring the absorbance at 470 nm.

### 2.3. Reduced (GSH) and Oxidized (GSSG) Glutathione Contents

For glutathione determination, the cellular pellet from 400 mL of algal culture was resuspended in 5 mL of extraction buffer (5% sulfosalicylic acid). Cells were lysed by passing twice at 1100 p.s.i. through a French pressure cell. The GSH and GSSG contents were determined as previously described by Salbitani et al. [20] for *C. sorokiniana*. Crude extract (100 µL) was added to 600 µL of reaction buffer (0.1-M Na-phosphate, pH 7.00 and 1-mM of ethylenediaminetetraacetic acid (EDTA), 40 µL of 0.4% 5,5′-dithiobis-(2-nitrobenzoic acid) (DTNB) (5,5′-dithiobis-2-nitrobenzoic acid), and 400 µL of distilled water. The GSH content was determined at 412 nm after 5 min. Then, 50 µL of 0.4% reduced nicotinamide adenine dinucleotide phosphate (NADPH) and 1 µL of glutathione reductase (GR) (0.5 U) were added to the reaction mixture, and the content of total glutathione (GSH plus GSSG) was determined at 412 nm after 30 min of incubation at room temperature.

### 2.4. Antioxidant Activity Determinations

Algal cultures (200 mL) were harvested by low-speed centrifugation (4500× *g* for 8 min), resuspended in 3 mL of cold extraction buffer (50-mM phosphate buffer, pH 7.5), and broken by passing twice through a French pressure cell (1100 psi). The homogenate was centrifuged at 12,000× *g* for 30 min at 4 °C, and the clear supernatant was used as a crude extract.

According to the method of Re et al. [21] and subsequent modifications by Bottone et al. [22], antioxidant activities were determined following the decolorization of the ABTS (2,29-azinobis-(3-ethylbenzothiazoline-6-sulfonic acid)) at 734 nm. For this study, ascorbic acid (Asa) was used as antioxidant standard (0–20 µM).

### 2.5. Animals

Sixty-day-old male Wistar rats, supplied by Envigo (Correzzana, Italy), at day 45 of age were used for the experiments. All animals were fed the same diet, a commercial rat chow purchased from Envigo, Italy and water from an ad libitum basis, and were kept two per cage under a constant artificial circadian cycle of 12 h of light and 12 h of darkness and 50% ± 10% relative humidity. From day 50, animals were randomly assigned to one of two dietary regimens, receiving either the control diet or a *Chlorella Sorokiniana* (CS)-supplemented diet. The latter consisted of the commercial rat chow to which the dried algae was added to a final concentration of 10 g/Kg, and both diets were administered for 10 days. One-half of the animals on both the control and supplemented diets at 50 days were subjected to treatment with 3,5,3′ triiodothyronine (T_3_), receiving a daily, intraperitoneal (i.p.) dose of 10 µgT_3_/100 g of their body weight.

The treatment of animals in these experiments was performed according to the guidelines set forth by the University’s Animal Care Review Committee (approval number 765/2016-PR). This treatment induced moderate hyperthyroidism, a condition that is associated to mild lysis of hepatic cells not significant enough to alter liver function indices (serum alanine transaminase, aspartate aminotransaminase, and alanine phosphatase) [23].

At the end of the treatments, the animals were anesthetized and sacrificed by beheading. The liver was immediately excised and placed into ice-cold homogenization medium (HM) (220-mM mannitol, 70-mM sucrose, 1-mM EDTA, 0.1% fatty acid-free albumin, and 10-mM Tris, pH 7.4), freed from superficial connective tissue, weighed, finely minced, and washed with HM. The tissue was gently homogenized (20% weight: volume) in HM by a glass Potter-Elvehjem homogenizer (Heidolph Instruments, Schwabach Germany) (500 rpm for 1 min). Tissue homogenates were used for analytical procedures.

### 2.6. Preparation of Intact Mitochondria

The homogenates, diluted 1:1 with HM, were centrifuged at 500× *g* for 10 min at 4 °C to remove debris and nuclei, and, subsequently, the supernatants were centrifuged at 10,000× *g* for 10 min. The mitochondrial pellets were resuspended in washing buffer (WB) (220-mM mannitol, 70-mM sucrose, 1-mM ethylene glycol-bis(β-aminoethyl ether)-*N,N,N*′,N′-tetraacetic acid (EGTA), and 20-mM Tris, pH 7.4) and centrifuged again at 10,000× *g* for 10 min. This step was repeated to wash intact mitochondria before a final suspension in WB. Mitochondrial protein content was measured by the biuret method [24].

### 2.7. Oxidative Damage and In Vitro Susceptibility to Oxidant

The level of lipid hydroperoxides (HP) were used to measure the extent of the lipid peroxidative processes in tissue homogenates and mitochondria according to Heath and Tappel [25] and expressed as n moles of NADPH consumed per min per g of tissue or per mg of mitochondrial proteins. The level of protein-bound (CO) carbonyl was used to determine protein oxidative damage to homogenates and mitochondria according to the procedure of Reznick and Packer [26] and the modified procedure of Schild et al. [27], respectively. Protein carbonyl content is expressed as n moles of 2,4-dinitrophenylhydrazine (DNPH) bound to carbonyl group per mg of proteins.

Susceptibility to oxidative stress of hepatic tissue was evaluated by the change in hydroperoxide levels following the treatment of 10% tissue homogenate with iron and ascorbate (Fe/As) at a concentration of 100/1000 μM for 10 min at room temperature. The reaction was blocked by adding 0.2% 2, 6-di-t-butyl-p-cresol (BHT), and the hydroperoxide levels were evaluated as previously described.

### 2.8. Oxygen Consumption and Cytochrome Oxidase (COX) Activity

The rates of oxygen consumption in tissue homogenates and mitochondria were determined at 30° using an Hansatech respirometer in 1.0 mL of incubation medium (145-mM KCl, 30-mM Hepes, 5-mM KH_2_PO_4_, 3-mM MgCl_2_, and 0.1-mM EGTA, pH 7.4), with 50 μL of 20% (*w*/*v*) homogenate or 0.25 mg of mitochondrial protein per mL. We used succinate (10 mM), plus 5-μM rotenone or pyruvate/malate (10/2.5 mM), as substrates in the absence (State 4) and in the presence (State 3) of 500-μM adenosine diphosphate (ADP). State 4 oxygen consumption of homogenates was measured adding 2 μg/mL of oligomycin.

Liver homogenates and mitochondrial suspensions were diluted with modified Chappel-Perry medium to have per mL either 100 mg of tissue or 0.2 mg of mitochondrial proteins and were used to determine cytochrome oxidase (COX) activity by the procedure of Barré et al. [28].

### 2.9. Mitochondrial H_2_O_2_ Release

The increase in fluorescence (Ex_wave_ 320 nm, Em_wave_ 400 nm) due to *p*-hydroxyphenylacetate (PHPA) oxidation by H_2_O_2_ in the presence of horseradish peroxidase (HRP) [29] in a Jasko fluorometer (Jasko, Pfungstadt, Germany) thermostatically controlled (30 °C) was used to determine the rate of mitochondrial H_2_O_2_ release during respiration. Mitochondrial proteins, 0.1 mg/mL of, HRP, 6 U/mL, and PHPA, 200 μg/mL, were added to a buffer solution containing 145-mM KCl, 30-mM Hepes, 5-mM KH_2_PO_4_, 3-mM MgCl_2_, and 0.1-mM EGTA, pH 7.4. After 30 s of stabilization, 10-mM succinate plus 5-μM rotenone or 10-mM pyruvate plus 2.5-mM malate were added as respiratory substrates to start the H_2_O_2_ release during State 4 of respiration. The subsequent addition of 500-μM ADP allowed to measure H_2_O_2_ release during State 3 of respiration. Furthermore, the effects of two electron chain inhibitors were investigated: rotenone (Rot), which blocks the transfer of electrons from Complex I to ubiquinone [30], and antimycin A (AA), which interrupts electron transfer within the ubiquinone-cytochrome b site of Complex III [31]. The concentration of the inhibitors (5-μM Rot and 10-μM AA) do not interfere with the detection PHPA-HRP system [32].

### 2.10. Analysis of Transcription Factors

Western Blot analysis was performed to evaluate the levels of expression of PGC-1, NRF-1, and NRF-2. Fragments of liver were homogenized (1:10, *w/v*) in a buffer containing 500-mM NaCl, 0.5% nonidet P-40, 6-mM EDTA, 6-mM EGTA, 1-mM dithiotreitol, and 40-mM Tris-HCl, pH 8.0, with an antiprotease mixture including 40-μg/mL phenylmethylsulfonyl fluoride (PMSF), 5-μg/mL leupeptin, 5-g/mL aprotinin, and 7-g/mL pepstatin. The supernatants obtained by the centrifugation of homogenates at 1000× *g* for 10 min at 4 °C were used for the preparation of the samples as described [33]. In brief, 5 μL of 3% SDS, 30% glycerol, 15% β-mercaptoethanol, 0.1% bromophenol blue, and 0.187-M Tris base, pH 6.8 were added to 10 μL of supernatant containing 1.5 mg/mL of proteins. The samples were boiled for 5 min, loaded on the gel, and electrophoresed [33]. Gel was run in the mini protean equipment (Bio-Rad laboratories Hercules, California, USA) for about 1 h at constant voltage (25 V). The hepatic proteins were transferred by electroblotting to polyvinylidene difluoride (PVDF) membrane. The membranes were incubated overnight at 4 °C, with 1:1000 antibodies to PGC-1 (SC-13067), NRF-1 (SC-33771), and NRF-2 (SC-22810) (Santa Cruz Biotechnology, Santa Cruz, CA, USA) in 154-mM NaCl, 10-mM Tris-HCl, pH 8.0, 2.5% nonfat dry milk, and 10% Tween 20 (blocking solution). The rabbit polyclonal antibodies we used, were raised against amino acids 1–300 mapping near the N-terminus of PGC-1 (which is common to the different isoforms of the coactivator), 204–503 mapping at the C-terminus of NRF-1, and 1–180 mapping near the N-terminus of NRF-2. The incubation with a 1:5000 secondary antibody (1 h at 37 °C), peroxidase-conjugated anti-first IgG antibodies (Santa Cruz Biotechnology), was used for the revelation of the binding with the primary antibody by chemiluminescence (ECL; Santa Cruz Biotechnology). The blots were stripped with 0.2-M NaOH, washed, and again incubated in blocking solution. Then, the blots were incubated for 2 h with 1:2000 anti-actin antibody, treated with secondary antibody, and used for the ECL revelation. Actin was used for the standardization. To compare protein expression levels among groups, a standard control sample was run on each gel, and all group values were then compared with such a sample that was assigned a value of 1.

### 2.11. Antioxidant Enzymes

Glutathione peroxidase (GPX) and glutathione reductase (GR) activities were assayed following the spectrophotometric reduction of NADPH absorbance at 340 nm. The former was performed at 37 °C according to Flohé and Günzler [34], with H_2_O_2_ as a substrate; the latter was measured at 30 °C according to Carlberg and Mannervik [35].

### 2.12. Data Analysis

The data were expressed as the means ± standard errors and analyzed by the two-way analysis of variance method. When a significant F ratio was found, the Bonferroni test was used to determine the statistical significance between means. Probability values (*p*) < 0.05 were considered significant.

## 3. Results

### 3.1. Chlorella Sorokiniana

*Chlorella sorokiniana* 211/8K grown under bicarbonate supplementation (1 g/L), at the late-exponential phase, reached: (i) a cell number of 6.9 × 10^6^ ± 0.175 cell/mL, (ii) a cell size of 3.11 ± 0.39 nm, and (iii) a biomass of 0.25 g/L dry weight.

In Table 1, the pigment content, total glutathione, GSH, and antioxidant capacities are reported.

### 3.2. Body Parameters

Thyroid hormone treatment did not change the body weight (334 ± 4.7, 324 ± 10, 306 ± 13.2, and 323 ± 6.9 for C, CS, T_3_, and T_3_S rats, respectively) but determined an increase in heart weight (0.79 ± 0.02, 0.73 ± 0.03, 0.89 ± 0.04, and 0.89 ± 0.05 for C, CS, T_3_, and T_3_S rats, respectively). Therefore, the heart weight/body weight ratio (2.35 ± 0.05, 2.27 ± 0.06, 2.83 ± 0.17, and 2.74 ± 0.15 for C, CS, T_3_, and T_3_S rats, respectively) increased in hyperthyroid animals independently from alga supplementation.

### 3.3. Oxidative Damage

**Figure 1:**
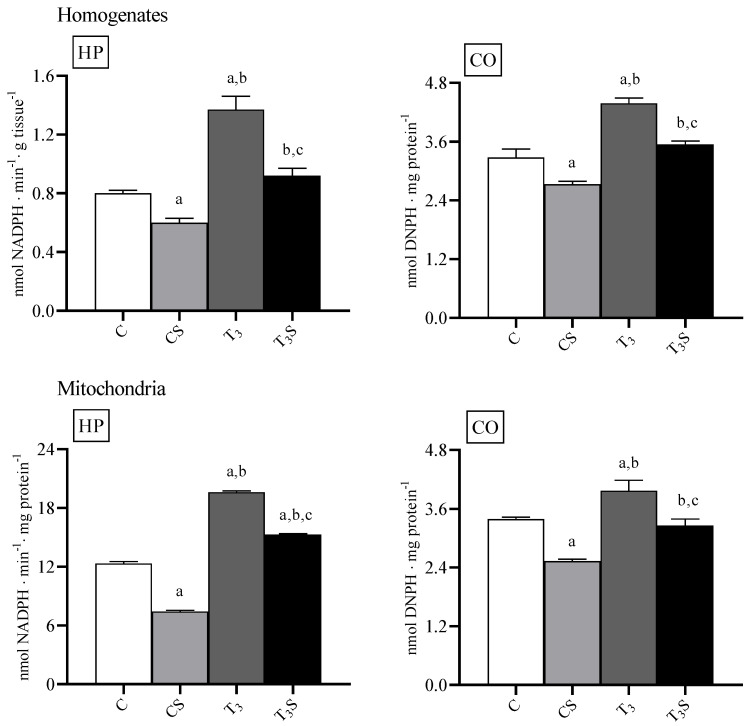
Levels of oxidative stress markers, lipid peroxides (HP), and protein carbonyl (CO) in rat liver homogenates and mitochondria from: C, control rats; CS, control rats fed with *Chlorella sorokiniana*; T_3_, rats made hyperthyroid by ip. T_3_ administration; and T_3_S, hyperthyroid rats fed with *Chlorella sorokiniana*. The values are the means ± SEM of the data obtained from eight different livers. ^a^ significant vs. C, ^b^ significant vs. CS, and ^c^ significant vs. T_3_. The level of significance was chosen as *p* < 0.05.

In Figure 1, the levels of lipid hydroperoxides in homogenate and mitochondria are reported. The level of lipid hydroperoxides were increased by the T_3_ treatment. Diet supplementation with *C. sorokiniana* reduced such levels in the homogenates and mitochondria of both control and T_3_-treated rats.

The level of protein carbonyls was increased by the T_3_ treatment in homogenates and mitochondria. *C. sorokiniana* diet supplementation reduced such levels in the homogenates and mitochondria of both control and T_3_-treated rats.

### 3.4. Oxygen Consumption and Cytochrome Oxidase Activity

**Figure 2:**
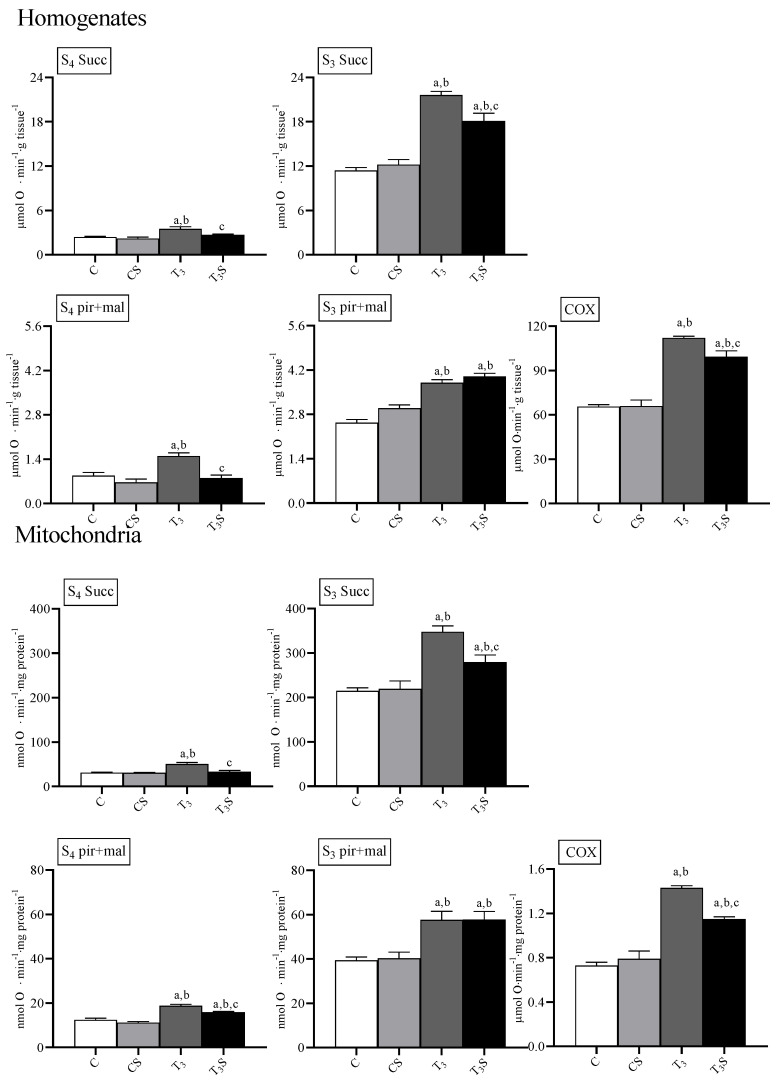
Rates of O_2_ consumption and cytochrome oxidase (COX) activity in rat liver homogenates and mitochondria from: C, control rats; CS, control rats fed with *Chlorella sorokiniana*; T_3_, rats made hyperthyroid by ip. T_3_ administration; and T_3_S, hyperthyroid rats fed with *Chlorella sorokiniana*. Rates of O_2_ consumption were measured in the absence (State 4, S_4_) and in the presence (State 3, S_3_) of ADP with Complex II (succinate, succ) and Complex I (pyruvate plus malate, pyr + mal)-linked substrates. The values are means ± S.E.M of the data obtained from eight different livers. ^a^ significant vs. C, ^b^ significant vs. CS, and ^c^ significant vs. T_3_. The level of significance was chosen as *p* < 0.05.

In Figure 2, the rates of oxygen consumption and the activities of the enzyme cytochrome oxidase are reported.

T_3_ treatment increased the rate of oxygen consumption in homogenates and mitochondria during State 4 and State 3 of respiration with both succinate and the mix pyruvate plus malate as respiratory substrates, according to its effects on the metabolic capacities in the target tissues [13,14].

In the homogenates, the diet supplementation with *C. sorokiniana* reduced the O_2_ consumption rate in T_3_-treated animals during the State 4 of respiration with both respiratory substrates and during the State 3 of respiration with the succinate.

In mitochondria, diet supplementation with *C. sorokiniana* reduced the O_2_ consumption rate only during the State 4 of respiration with both succinate and pyruvate plus malate and during the State 3 of respiration with succinate as respiratory substrates.

In homogenates, respiratory control ratios (RCRs) with succinate were 4.5 ± 0.21, 4.9 ± 0.23, 6.2 ± 0.21, and 5.5 ± 0.03 in C, Cs, T_3_, and T_3_S, respectively. Hyperthyroidism increased RCR values independently from algal supplementation. With pyruvate plus malate, RCRs were 3.5 ± 0.11, 3.8 ± 0.18, 2.9 ± 0.10, and 4.9 ± 0.04, in C, Cs, T_3_, and T_3_S, respectively. T_3_ treatment decreased RCR. Alga supplementation increased RCR in T_3_-treated animals.

In mitochondria, RCRs with succinate were 7.2 ± 0.09, 7.1 ± 0.14, 6.4 ± 0.09, and 7.7 ± 0.16 in C, Cs, T_3_, and T_3_S, respectively. T_3_ treatment decreased the RCR. Alga supplementation increased the RCR in T_3_-treated animals. With pyruvate plus malate as respiratory substrates, RCRs were: 2.6 ± 0.11, 3.0 ± 0.17, 2.8 ± 0.06, and 3.0 ± 0.06 in C, Cs, T_3_, and T_3_S, respectively.

The RCRs values suggest that mitochondrial integrity was preserved during samples preparation.

The activity of the enzyme cytochrome oxidase was affected by treatments. T_3_ treatment increased the COX activity in homogenates and mitochondria. Diet supplementation with *C. sorokiniana* attenuated the T_3_-induced increase in COX activity in both homogenates and mitochondria.

### 3.5. Mitochondrial ROS Release

**Figure 3:**
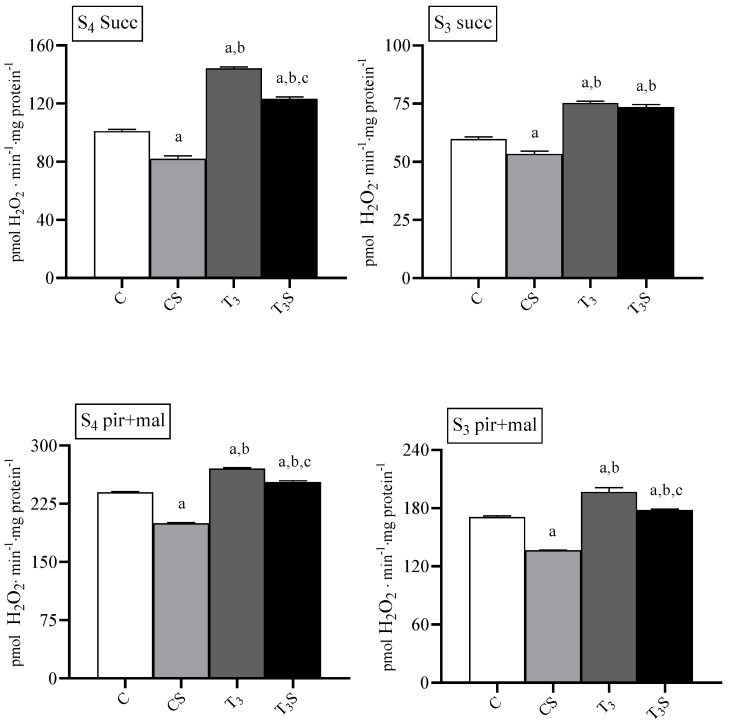
Rates of H_2_O_2_ release in rat liver homogenates and mitochondria from: C, control rats; CS, control rats fed with *Chlorella sorokiniana*; T_3_, rats made hyperthyroid by ip. T_3_ administration; and T_3_S, hyperthyroid rats fed with *Chlorella sorokiniana*. Rates of H_2_O_2_ release were measured in the absence (State 4, S_4_) and in the presence (State 3, S_3_) of ADP with Complex II (succinate, succ) and Complex I (pyruvate plus malate, pyr + mal)-linked substrates. The values are the means ± S.E.M of the data obtained on eight different livers. For each value, eight rats were used. ^a^ significant vs. C, ^b^ significant vs. CS, and ^c^ significant vs. T_3_. The level of significance was chosen as *p* < 0.05.

Mitochondrial ROS release was increased by T_3_ treatment during basal and ADP-stimulated respiration with both respiratory substrates (Figure 3). In mitochondria from control rats, the diet supplementation with *C. sorokiniana* reduced the H_2_O_2_ release during basal and ADP-stimulated respiration.

In mitochondria from T_3_-treated rats *C. sorokiniana* reduced mitochondrial H_2_O_2_ the release with succinate only during basal respiration and with pyruvate plus malate during both basal and ADP-stimulated respiration.

### 3.6. Effect of Inhibitors on Mitochondrial H_2_O_2_ Release

The effects of inhibitors of the respiratory chain are reported in Table 2. In the presence of succinate, H_2_O_2_ release rates were increased by T_3_ treatment and decreased by dietary alga supplementation. The addition of Rot, which blocks the transfer of electrons from Complex I to ubiquinone [30], to mitochondria respiring in the presence of succinate decreased the H_2_O_2_ release rates, which remained higher in T_3_-treated and lower in alga-supplemented rats. The addition of AA, which interrupts electron transfer within the ubiquinone-cytochrome b site of Complex III [31], further increased the H_2_O_2_ release rates, which remained higher in T_3_-treated rats and lower in *C. sorokiniana*-supplemented animals.

In the presence of pyruvate plus malate, the rates of H_2_O_2_ release were increased by the T_3_ treatment and reduced by *C. sorokiniana* supplementation. The addition of AA or Rot to pyruvate plus malate respiring mitochondria increased the H_2_O_2_ release rates, which were higher in T_3_-treated animals than in control animals. *C. sorokiniana* reduced the H_2_O_2_ release rate in the presence of AA in the control but not in T_3_-treated animals, while reduced the H_2_O_2_ release rate in both the control and T_3_-treated animals in the presence of rotenone.

### 3.7. Mitochondrial Transcription Factors

**Figure 4:**
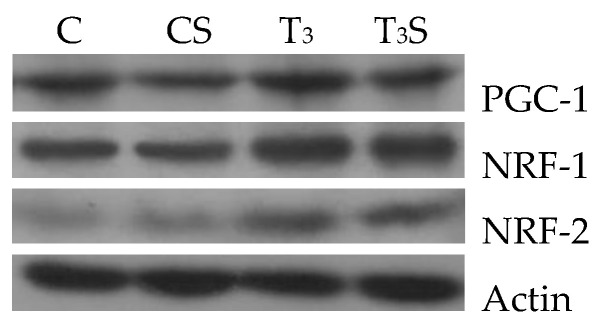

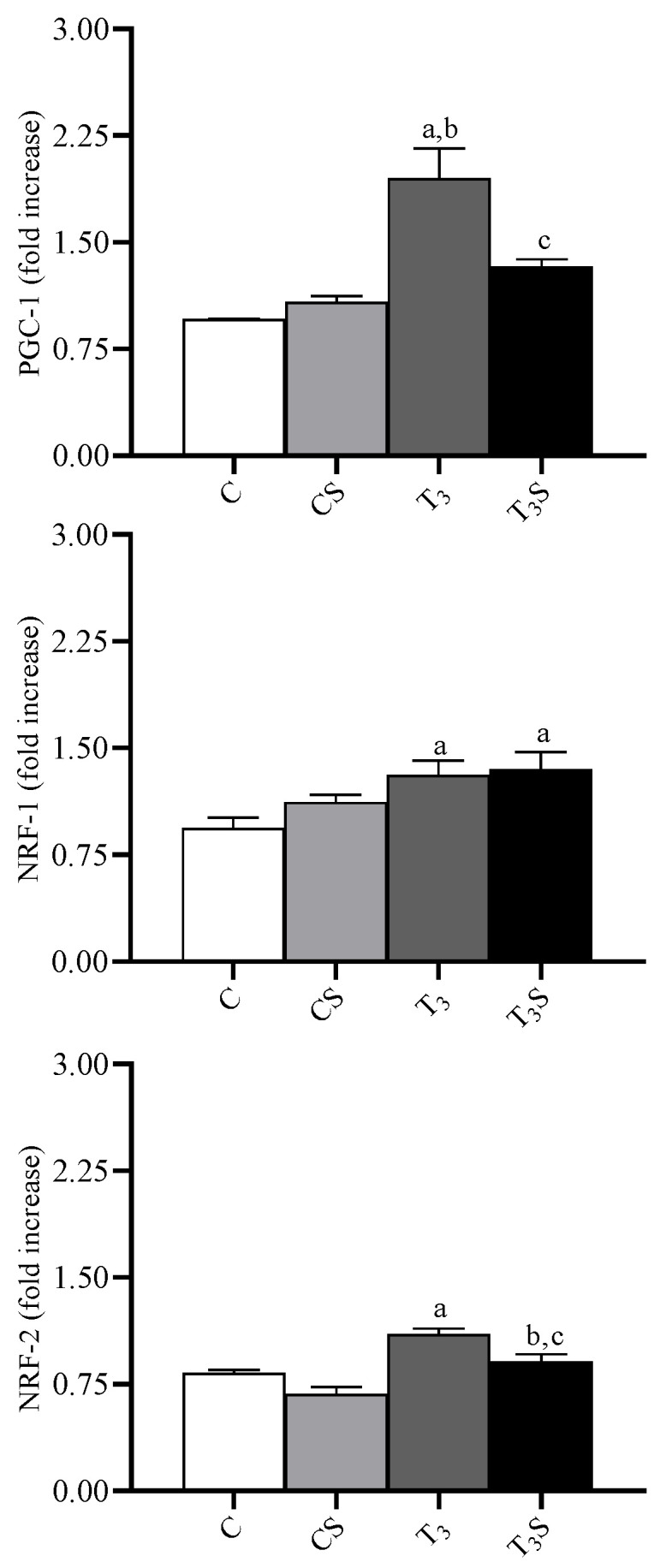
Levels of peroxisomal proliferator-activated receptor-γ coactivator (PGC-1), nuclear respiratory factor 1 (NRF-1), and nuclear respiratory factor 2 (NRF-2) protein expressions in rat livers. Liver total proteins from hypothyroid from C, control rats; CS, control rats fed with *Chlorella sorokiniana*; T_3_, rats made hyperthyroid by ip. T_3_ administration; and T_3_S, hyperthyroid rats fed with *Chlorella sorokiniana* were isolated and analyzed using Western blot analyses. A representative blot of PGC-1, NRF-1, NRF-2, and actin protein expressions is shown (above). The values reported in the graphs are the means ± S.E.M. of three independent experiments performed on three different livers. Ratios of band intensities to the β-actin band intensities were compared with a standard control sample that was assigned a value of 1. ^a^ significant vs. C, ^b^ significant vs. CS, and ^c^ significant vs. T_3_. The level of significance was chosen as *p* < 0.05.

In Figure 4, the changes in the liver tissue levels of the transcription factors PGC-1, NRF-1, and NRF-2 and their representative images are reported. The levels are increased by the T_3_ treatment. Alga dietary supplementation prevented the increase in the levels of PGC-1 and of NRF-2 in hyperthyroid animals.

### 3.8. Antioxidant Enzyme Activities

**Figure 5:**
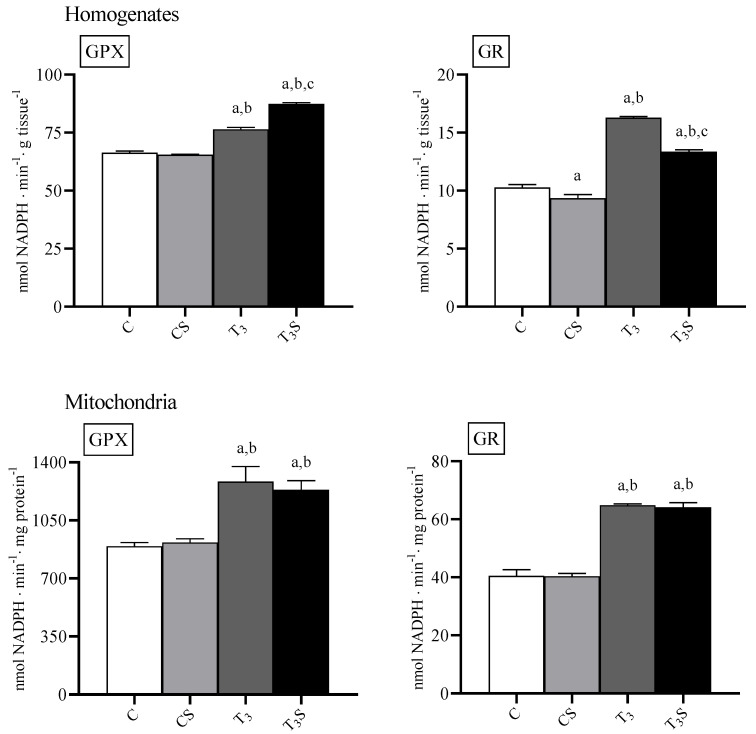
Activity of the antioxidant enzymes glutathione peroxidase (GPX) and glutathione reductase (GR) in rat liver homogenates and mitochondria from: C, control rats; CS, control rats fed with *Chlorella sorokiniana*; T_3_, rats made hyperthyroid by ip. T_3_ administration; and T_3_S, hyperthyroid rats fed with *Chlorella sorokiniana*. The values are the means ± S.E.M of the data obtained on eight different livers. ^a^ significant vs. C, ^b^ significant vs. CS, and ^c^ significant vs. T_3_. The level of significance was chosen as *p* < 0.05.

In Figure 5, the activities of the antioxidant enzymes GPX and GR are reported. In homogenates, the T_3_ treatment increased both the GPX and GR activity. In the liver of hyperthyroid animals, dietary *C. sorokiniana* supplementations further increased the GPX activity but reduced the GR activity.

In mitochondria, the T_3_ treatment increased both the GPX and GR activity, but the *C. sorokiniana* dietary supplementation did not influence the enzyme activities.

### 3.9. In Vitro Susceptibility to Oxidants in Homogenates and Mitochondria

**Figure 6:**
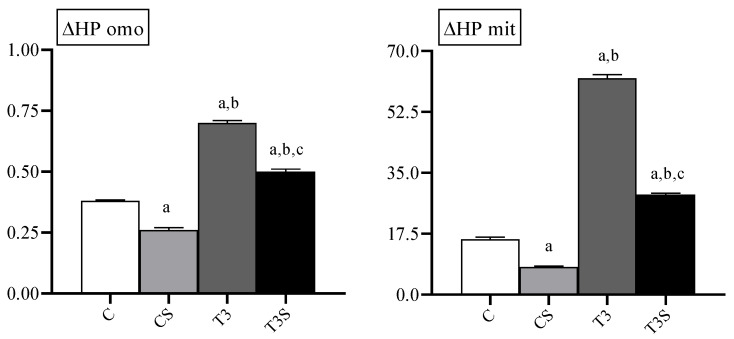
Susceptibility to oxidative stress in rat liver homogenates (omo) and mitochondria (mit) from C, control rats; CS, control rats fed with *Chlorella sorokiniana*; T_3_, rats made hyperthyroid by ip. T_3_ administration; and T_3_S, hyperthyroid rats fed with *Chlorella sorokiniana.* Susceptibility to oxidative stress was evaluated by changes in the HP levels (ΔHP) after Fe/As treatment. The values are means ± S.E.M of the data obtained on eight different livers. ^a^ significant vs. C, ^b^ significant vs. CS, and ^c^ significant vs. T_3_. The level of significance was chosen as *p* < 0.05.

In Figure 6, the changes in the levels of lipid-bound hydroperoxides after in vitro Fe^2+^-ascorbate-induced oxidative stress are reported. In both the homogenates and mitochondria of control and hyperthyroid animals, the susceptibility to oxidative stress was increased by the T_3_ treatment and reduced by alga supplementation.

## 4. Discussion

In the present study, we show that the dietary supplementation with *Chlorella sorokiniana* reduces the levels of the markers of oxidative stress in the liver of both control and hyperthyroid animals. Our results suggest that the alga can interfere with cellular ROS production, removal, or both processes.

The reduction of the levels of the oxidative damage markers in the control animals is not surprising if we consider that the tissues always show a low level of oxidative damage probably dependent on the not-perfect balancing between ROS generation and antioxidants capacities [36]. In the hyperthyroid animals, the increased levels of the lipid and protein oxidation are linked mainly to the metabolic effects of the hormone [1]. The thyroid hormone increases the metabolic capacities of the target tissues, increasing the mitochondrial electron carrier content [1], and this leads to the increase in mitochondrial ROS production and oxidative damage [13].

Here, we report that *C. sorokiniana* reduces the mitochondrial oxidative damage to both lipids and proteins. Such effects parallel the reduction in the state 4 oxygen consumption in both liver homogenate and mitochondria. The state 4 oxygen consumption rate depends on the degree of the inducible and basal uncoupling of the inner mitochondrial membrane [37]. Several experimental pieces of evidence show that the basal proton conductance is affected by phospholipid oxidation. In membrane model systems, lipid oxidation causes an increase in the proton leak, a decrease in electrical resistance, and a decrease in membrane thickness [37]. Furthermore, the induction of lipid oxidation in brain mitochondria by peroxynitrite (product by the reaction between superoxide and nitric oxide) causes an increase in the proton leak that is prevented by the water-soluble antioxidant Trolox [38]. Therefore, it is possible to hypothesize that the alga dietary supplementation protects inner membrane components by oxidation, reducing the proton leak.

*Chlorella sorokiniana* dietary supplementation also affects the thyroid hormone-induced increase in state 3 respiration. We find that the ADP-stimulated oxygen consumption is increased by hyperthyroidism regardless of algae supplementation, but, in both liver homogenates and mitochondria, the thyroid hormone effect appears attenuated when oxygen consumption is determined using succinate as a respiratory substrate. This suggests that alga can affect the thyroid hormone-induced changes in the mitochondrial population. The determination of the enzyme cytochrome oxidase activity that is correlated to the maximal aerobic capacity [39] shows that the alga attenuates the thyroid hormone-induced increase of the aerobic capacities of the hepatic tissue and mitochondria. The increased tissue aerobic capacity can depend on changes in the tissue content of mitochondria or on the increase in their aerobic capacity. The ratio between the activities of the enzyme cytochrome oxidase in the tissue and mitochondria furnishes a rough evaluation of the mitochondrial mass in the tissue (mg of mitochondrial protein/g tissue). We find that such a ratio (89.94 ± 3.01, 83.49 ± 9.33, 78.51 ± 3.37, and 85.92 ± 3.34 for C, Cs, T_3_, and T_3_S, respectively) is not changed by the thyroid hormone treatment, according to previous observations showing that, in experimental hyperthyroidism, the increase in hepatic respiration involves increases in the number of respiratory chain proteins and inner surface area of the mitochondria [40] without changes in their number [41] and protein mass [42]. Alga administration does not affect the mitochondrial protein mass but affects other characteristics of the mitochondrial population, as shown by the analysis of the effects of the alga on the mitochondrial release of hydrogen peroxide. The mitochondrial respiratory chain is one of the most important, if not the main, cellular ROS sources [1]. It has been shown that, along the mitochondrial chain, some electron carriers undergo autoxidation by transferring the electron to oxygen instead of to the next carrier. The primary ROS generated within mitochondria by the univalent autoxidation is the superoxide, which is converted by the mitochondrial superoxide dismutase enzyme into hydrogen peroxide, which can be turned into hydroxyl radical via the Fenton reaction [1].

The main sites involved in mitochondrial ROS production are localized at Complexes I and III [43]. However, succinate-dependent ROS production by Complex II from rat skeletal muscle [43] and glycerol 3-phosphate-dependent production by Complex II from several rat tissues [44] have also been reported. It is now clear that most of the hydrogen peroxide produced in the mitochondria is removed by the efficient antioxidant defences system of the mitochondria and that it is possible to determine the hydrogen peroxide released but not that produced [45]. We evaluated the mitochondrial hydrogen peroxide release using a combination of malate/pyruvate to generate a matrix NADH for respiration by Complexes I, II, and IV. The pyruvate is oxidized by pyruvate dehydrogenase, generating NADH and acetyl-CoA, which further react with the matrix oxaloacetate to shift the malate dehydrogenase reaction towards the oxidation of malate and formation of NADH. Succinate provides respiration via Complexes II, III, and IV. The succinate was used in combination with rotenone to block the inverse flux of electrons from Complex III to Complex I. In hyperthyroid animals, the increased metabolism associates with an increased hydrogen peroxide release in liver mitochondria during both basal (State 4) and ADP-stimulated respiration (State 3), according to what was previously found [42]. *C. sorokiniana* dietary integration not only reduces the thyroid hormone-induced increase in hydrogen peroxide release but, also, the release rates in the control animals. To obtain more information on the effects of the alga on the mitochondrial respiratory chain, we measured the hydrogen peroxide release in the presence of respiratory chain inhibitors. It has been shown that the mitochondrial ROS production depends on the content and the reduction degree of the autoxidizable electron carriers in the inner mitochondrial membrane [45]. Stopping the electron flux with respiratory inhibitors makes the electron chain carriers before the block site completely reduced. In such a condition, ROS release depends only on the content of the autoxidizable carriers before the block. We used adequate combinations of respiratory substrates and inhibitors to obtain information about the content of the autoxidizable carriers. The addition of antimycin A to mitochondria during succinate supported respiration in the presence of rotenone and provided information on the content of the autoxidizable carrier located at complex III. Furthermore, the addition of antimycin A to mitochondria during pyruvate plus malate-supported respiration provides information on the contents of both the autoxidizable carriers located at complexes I and III. Finally, the addition of rotenone to mitochondria respiring in the presence of pyruvate plus malate furnishes information on the contents of the autoxidizable carrier located at complex I. According to what was previously reported, the T_3_ treatment increases the contents of both autoxidizable carriers in complexes I and III [2]. Alga dietary supplementation seems to reduce the content of the autoxidizable electron carriers both at complexes I and III in the control and hyperthyroid animals, an effect that does not appear linked to changes in the expression of the factors involved in mitochondrial biogenesis.

Nuclear respiratory factors 1 and 2 (NRF-1 and NRF-2) and the peroxisomal proliferator-activated receptor-γ coactivator (PGC-1) are nuclear regulatory proteins that control the expression of the respiratory apparatus. NRF-1 and NRF-2 are transcriptional factors linked to the transcription of many genes involved in mitochondrial functions and biogenesis [46]. PGC-1 is a transcriptional coactivator involved in the control of the respiratory apparatus as an intermediary between environmental stimuli and transcriptional responses [46]. It has been reported that T_3_ triggers processes, such as mitochondrial biogenesis, adaptive thermogenesis, and hepatic gluconeogenesis [47], which resemble those regulated by PGC-1, which, in turn, interacts with several nuclear hormone receptors, including thyroid hormone receptor β [48]. Significant increases in PGC-1 protein levels after T_3_ treatment have been found in a variety of tissues, including the liver [49]. Although some data indicate that thyroid hormone-mediated gene expression patterns are not completely dependent on PGC-1 activation [50], the above data support the idea that a thyroid hormone-mediated activation of PGC-1 might help this coactivator to exert its function in the adaptation to endocrine signals. Our data show that the dietary supplementation with the alga prevents the thyroid hormone-induced increase in PGC-1 and NRF-2 protein levels. These effects are also associated with a lower increase in the maximal aerobic capacities, as suggested by the changes in the activity of the cytochrome oxidase in alga-fed animals. It is possible to hypothesize that the lower PGC-1 expression can be due to the lower ROS production in alga-fed animals. Indeed, it has been reported that PGC-1 expression can be upregulated by ROS [51] and that most of the transcriptional factors are under ROS regulation [52,53]. The observation that alga supplementation reduces lipid and protein oxidation in control rats without modifying PGC-1 and NRF-2 suggests that alga protection could not only be due to the reduction in ROS production but, also, to the increased effectiveness of the antioxidant defence system.

To obtain information on the changes in the efficacy of the antioxidant system, we determined the activity of two of the most important antioxidant enzymes, glutathione peroxidase and reductase. Their activity is increased by hyperthyroidism in both the liver homogenate and mitochondria and slightly changed by the dietary alga supplementation in the tissue homogenates only. The changes in the activity of the antioxidant enzymes and/or in the contents of the low molecular weight antioxidants do not furnish a complete picture of the effects of treatments on the capacity of the tissues to counteract an oxidative insult. Therefore, to obtain information about the effects of the alga supplementation on the antioxidant capacities, we determined the in vitro susceptibility to ferrous/ascorbate-induced oxidative stress. The alga supplementation significantly reduces the in vitro susceptibility to oxidants, mainly in the mitochondrial fraction. This suggests that a dietetic integration with *Chlorella sorokiniana* can be useful in the prevention of the disfunction in which mitochondrial oxidative damage and ROS production play a putative role, such as the insulin resistance [54] that preludes the onset of type 2 diabetes. *C. sorokiniana* contains fat-soluble antioxidants such as α-tocopherol and carotenoids. These latter are in high amounts and are mainly constituted by lutein, for about 60%. Dietary carotenoids are absorbed and accumulated in the liver and other organs, where they exert their beneficial effects. The intake of carotenoids can significantly reduce the risk of suffering from liver diseases, such as nonalcoholic fatty liver disease [55]. Moreover, it has been shown that the consumption of wolfberry, which contains high amount of lutein, attenuates retinal mitochondrial stress [56]. These observations suggest that the carotenoids, particularly lutein, can protect liver mitochondria from oxidative insult. On the other side, *C. sorokiniana* also contains water-soluble antioxidants, among which the glutathione, and other fat-soluble antioxidants, among which α-tocopherol and the complex antioxidant composition of the alga, do not allow to identify a single substance responsible for its beneficial effect against liver oxidative damage. Thus, it is possible to hypothesize that the protective effect of *C. sorokiniana* could be ascribed to the synergic action of the different antioxidants it contains. In conclusion, our data suggest that dietary supplementation with *C. sorokiniana* can be useful in the protection of the liver in conditions in which oxidative stress develops.

## Figures and Tables

**Table 1:** Content of pigments and total and reduced glutathione and antioxidant capacity of *Chlorella sorokiniana.*

Chlorophyll-*a* (mg/g)	22.44 ± 0.64
Chlorophyll-*b* (mg/g)	19.20 ± 0.28
Carotenoids (mg/g)	10.32 ± 0.08
Total glutathione (μmol/g)	5.60 ± 0.08
GSH (μmol/g)	3.04 ± 0.16
ABTS (Equation nmol Asa/g)	87.68 ± 3.76

The values are the means ± SEM of three different cultivations. The chlorophyll-*a* (Chl-*a*) -*b* (Chl-*b*), total carotenoids, total and reduced glutathione (GSH), and antioxidant activity were measured spectrophotometrically. Antioxidant activity was determined by decolorization of 2,29-azinobis-(3-ethylbenzothiazoline-6-sulfonic acid (ABTS). Ascorbic acid (Asa) was used as antioxidant standard.

**Table 2:** Effect of respiratory inhibitors on H_2_O_2_ release by liver mitochondria from the control, control fed *Chlorella sorokiniana*, hyperthyroid, and hyperthyroid fed *Chlorella sorokiniana* rats.

Substrate and Additions	Group			
	C	CS	T_3_	T_3_S
Succinate (Succ)	154.7 ± 1.0	127.05 ± 0.7 ^a^	197.4 ± 4.2 ^a,b^	175.8±1.5 ^a,b,c^
Succ + Rot	100.0 ± 0.7 *	84.1 ± 1.4 ^a,^*	142.6 ± 1.3 ^a,b,^*	120.7±0.8,^a,b,c,^*
Succ + Rot +AA	813.3 ± 3.8 *	774.4 ± 7.6 ^a,^*	922.7 ± 8.0 ^a,b,^*	855.6±3.2 ^a,b,c,^*
Pyruvate/Malate (Pyr/Mal)	238.5 ± 1.6	196.4 ± 1.7 ^a^	270.5 ± 1.6 ^a,b^	253.4±2.2 ^a,b,c^
Pyr/Mal + AA	929.9 ± 6.6 *	847.0 ± 5.4 ^a,^*	1037.5 ± 19.5 ^a,b,^*	965.8±4.8 ^b,c,^*
Pyruvate/Malate	239.0 ± 1.1	198.2 ± 0.6 ^a^	269.2 ± 1.1 ^a,b^	251.9±0.8 ^a,b,c^
Pyr/mal + Rot	266.8 ± 1.8 *	249.4 ± 4.0 ^a,^*	291.2 ± 2.6 ^a,b,^*	281.0±1.1 ^a,b,c,^*

The values are the means ± SEM of the data obtained on eight different livers. One rat was used for each experiment. H_2_O_2_ release rates are expressed in pmol/min/mg protein. C, control rats; CS, control rats fed with *Chlorella sorokiniana*; T_3_, rats made hyperthyroid by ip. T_3_ administration; and T_3_S, hyperthyroid rats fed with *Chlorella sorokiniana.* Rot, rotenone and AA, Antimycin A. ^a^ significant vs. C rats, ^b^ significant vs. CS rats, and ^c^ significant vs. T_3_ rats; * significant effect of the last inhibitor added vs. mitochondria under same conditions without that inhibitor. The level of significance was chosen as *p* < 0.05.
